# The Characteristic of Muscle Function for Sarcopenia in Patients with Rheumatoid Arthritis: A Large-Scale Real-World Cross-Sectional Study

**DOI:** 10.3390/medicina61040551

**Published:** 2025-03-21

**Authors:** Pei-Wen Jia, Jian-Zi Lin, Yao-Wei Zou, Zhi-Ming Ouyang, Ying Yang, Kui-Min Yang, Liu-Hong Liang, Jin-Yuan Han, Ze-Hong Yang, Jian-Da Ma, Lie Dai

**Affiliations:** 1 Department of Rheumatology, Sun Yat-sen Memorial Hospital, Sun Yat-sen University, 107 Yan Jiang West Road, Guangzhou 510120, China; jiapw@mail2.sysu.edu.cn (P.-W.J.); linjz5@mail.sysu.edu.cn (J.-Z.L.); zouyw6@mail2.sysu.edu.cn (Y.-W.Z.); ouyzhm3@mail.sysu.edu.cn (Z.-M.O.); yangy935@mail2.sysu.edu.cn (Y.Y.); yangkm5@mail.sysu.edu.cn (K.-M.Y.); lianglh25@mail.sysu.edu.cn (L.-H.L.); hanjinyuan0220@163.com (J.-Y.H.); 2 Department of Radiology, Sun Yat-sen Memorial Hospital, Sun Yat-sen University, 107 Yan Jiang West Road, Guangzhou 510120, China; yangzeh2@mail.sysu.edu.cn

**Keywords:** rheumatoid arthritis, sarcopenia, grip strength, physical performance, functional limitation

## Abstract

*Background and Objectives*: Sarcopenia is a notable comorbidity of rheumatoid arthritis (RA), affecting about one third of patients. However, the characteristic of muscle function and its association with RA disease remains unknown. *Materials and Methods*: This cross-sectional study collected clinical data from a real-world Chinese RA cohort. Sarcopenia was defined as both myopenia and low muscle function (LMF). Myopenia was defined as appendicular skeletal muscle mass index (ASMI) < 7.0 kg/m^2^ in men and <5.7 kg/m^2^ in women. LMF was defined as low muscle strength (LMS, hand grip < 28 kg in men and <18 kg in women) or low physical performance (LPP, 6 m gait speed < 1.0 m/s). *Results*: Among 1125 RA patients recruited in this study, 928 RA patients were eligible for analysis. The prevalence of sarcopenia, myopenia, LMF, LMS, and LPP in all RA patients was 36.5%, 46.1%, 69.0%, 57.8%, and 37.1%, respectively. According to their trends in age and disease activity, there were 111 (11.9%) patients in the young (age < 50 years) and remission (CDAI ≤ 2.8) subgroup, 199 (21.4%) patients in the young and active (CDAI > 2.8) subgroup, 198 (21.3%) patients in the old (age ≥ 50 years) and remission subgroup, and 420 (45.2%) patients in the old and active subgroup. Compared with the two remission subgroups, respectively, the young and active subgroup had significantly lower grip strength, higher prevalence of sarcopenia, LMF, and LMS, and worse activity function. After adjustment for potential confounders, multivariate multinominal logistic regression analysis showed that the young and active subgroup was positively associated with sarcopenia (OR = 3.193, 95%CI: 1.477–6.899), LMF (OR = 2.390, 95%CI: 1.207–4.731), and LMS (OR = 3.520, 95%CI: 1.743–7.110). *Conclusions*: Worse muscle strength, rather than reduced physical performance, is more common in patients with active RA at a young age. It underscores the critical need for early identification and intervention of muscle dysfunction to improve their quality of life.

## 1. Introduction

Sarcopenia is a disease characterized by the age-associated loss of muscle mass and function [1]. The prevalence of sarcopenia among individuals with rheumatoid arthritis (RA) is approximately 33%, which is higher than the approximately 10% reported in the general population [2]. RA is an immune-mediated inflammatory disease consisting of erosive arthritis, accelerated musculoskeletal aging, and systemic organ involvement [3]. Unlike primary sarcopenia, which predominantly affects the elderly, RA-related sarcopenia can manifest in younger individuals. It is also characterized by pronounced muscle depletion, while fat mass remains stable or slightly increases in RA [4]. This condition differs from muscle wasting associated with chronic diseases like cancer cachexia and heart failure, which typically involve overall weight loss [5]. Though the exact causes of sarcopenia in RA are unclear, it is thought to result from a complex interplay of immunological and hormonal shifts, alongside cytokine activity of chronic inflammation, all of which contribute to muscle wasting and premature muscular aging [6].

A challenge in diagnosing and classifying sarcopenia within clinical and research settings arises from the diversity of criteria used [7]. In the last decade, various task forces, including the European Working Group on Sarcopenia in Older People (EWGSOP) and the Asian Working Group for Sarcopenia (AWGS), have developed consensus definitions for sarcopenia classification. In 2018, the EWGSOP emphasized poor muscle strength as a key feature of sarcopenia and established diagnostic criteria in the following order: low muscle strength, low muscle quantity or quality, and low physical performance [8]. In 2019, the AWGS defined sarcopenia as “age-related loss of muscle mass, plus low muscle strength, and/or low physical performance” [9]. Although tools have been developed to facilitate screening for sarcopenia in clinical settings, the widespread adoption of sarcopenia assessment in rheumatology clinical practices remains a challenge. Our previous series of real-world RA cohort studies showed that the prevalence of myopenia (low muscle mass) was 45.1% among all RA patients [10], 42.1% among early RA patients [11], and 54.5% among elderly RA patients [12]. Furthermore, our prospective studies indicated that baseline myopenia was an independent predictor of 1-year aggravated joint destruction in RA [13]. Other studies also indicated that low muscle mass was associated with higher disease activity score, severer radiographic damage, and falls in patients with established RA [14,15].

However, there is still a lack of studies on muscle function and its correlation with clinical characteristics in RA patients. Based on our large-scale real-world cross-sectional study in the RA cohort, we investigated the characteristics of muscle function among RA patients, identified clinical differences among subgroups with varying muscle function, and revealed the association between muscle function and RA disease.

## 2. Materials and Methods

### 2.1. Participants

Consecutive Chinese patients with RA aged ≥ 18 years who fulfilled the 2010 American College of Rheumatology (ACR)/European League Against Rheumatism (EULAR) [16] classification criteria for RA were recruited from November 2019 to September 2023 from a large-scale real-world cohort as described in our previous reports [10,11,12,13]. Exclusion criteria were as follows: with incomplete body composition and muscle function data, overlapping other autoimmune diseases (e.g., systemic lupus erythematosus, scleroderma, and dermatomyositis), serious infection, malignancy, and pregnancy. This study was conducted in compliance with the Declaration of Helsinki, and the protocol was approved by the Medical Ethics Committee of Sun Yat-Sen Memorial Hospital (SYSEC-KY-KS-2019-097). All participants gave their written informed consent before clinical data collection.

### 2.2. Demographic and Clinical Data Collection

The demographic and clinical data of patients with RA were recorded at enrollment, including gender, age, smoking history, disease duration, disease activity, radiographic indicators, activity function, comorbidities, and previous medications, as in our previous reports [10,11,12]. Disease activity defined by clinical disease activity index (CDAI) was divided into four categories: high disease activity (CDAI > 22), moderate disease activity (10 < CDAI ≤ 22), low disease activity (2.8 < CDAI ≤ 10), and remission (CDAI ≤ 2.8) [17]. Active disease was defined as CDAI > 2.8, including high, moderate, and low disease activity. Radiographic indicators included modified total Sharp score (mTSS), joint space narrowing (JSN) subscore, and joint erosion (JE) subscore [18].

Activity function was assessed using the Chinese language version of the Stanford Health Assessment Questionnaire (HAQ), which reflects difficulties in daily living and contains questions about the ability of patients to perform 20 activities of daily living, classified into 8 subdimensions with 2 or 3 activities in each subdimension. Eight subdimensions were divided into upper-limb (grip, reach, dressing, and eating) and lower-limb (walking, rising, hygiene, and usual activities) subdimensions [19]. The HAQ score ranges from 0 to 3, with higher scores indicating more severe dysfunction. Functional limitation was defined as a HAQ disability index (HAQ-DI) > 0, and functional limitation in each subdimension was defined as the score of that subdimension > 0 [20].

### 2.3. Body Composition

Anthropometric measurements of patients with RA were collected. Body composition (BC) was assessed by bioelectric impedance analysis using an InBody 230 device (Biospace Co., Shanghai, China) [10] without shoes, socks, bulky clothing, and other accessories. It measures BC indicators including weight, fat mass, body fat percentage, skeletal muscle mass, the mass and distribution of muscle and fat in trunk, and appendicular limbs. Height was measured to the nearest 0.01 m without shoes and socks using a stadiometer. BMI (kg/m^2^) was calculated as weight (kg) divided by height (m) squared. The appendicular skeletal muscle mass index (ASMI) was defined as appendicular skeletal muscle mass/height^2^ (kg/m^2^). Myopenia was defined by ASMI < 7.0 kg/m^2^ in men and <5.7 kg/m^2^ in women according to the AWGS 2019 [9].

### 2.4. Muscle Function

Sarcopenia was defined as both myopenia and low muscle function (LMF) [9]. Muscle function assessment included muscle strength and physical performance. For the measurement of grip strength as an indicator of muscle strength, we used a Smedley dynamometer in accordance with the AWGS 2019 recommendation. Patients stood with full elbow extension, were instructed to squeeze the dynamometer as hard as possible alternating three times for each hand, and the maximum value of the left or right hand was defined as the participant’s muscle strength. Low muscle strength (LMS) was defined by grip strength < 28 kg in men and <18 kg in women [9]. Physical performance was assessed with 6 m gait speed, in which patients were instructed to walk at their fastest pace along a 6 m course. Canes and other walking aids were allowed. The walk test was performed three times with the fastest time used for all analyses. Low physical performance (LPP) was defined by 6 m gait speed < 1.0 m/s, and LMF was defined as LMS or LPP [9]. According to muscle strength and physical performance, muscle function category was divided into four groups: normal, LMS only, LPP only, and both LMS and LPP.

### 2.5. Statistical Analysis

Statistical analyses were conducted by R (version 4.2.2) software. Data were summarized by means ± standard deviations (SD) or medians with interquartile range (IQR) for continuous variables according to the data distributions and the frequencies with percentages for categorical variables. Two independent samples t-test or Mann–Whitney U test was used to compare the differences in continuous variables according to distributions between two groups (male and female). One-way analysis of variance (ANOVA) or Kruskal–Wallis H analysis of variance on ranks was used for comparisons of the four groups according to their data distributions, such as comparisons among RA patients in four muscle function categorizations, and four subgroups according to age and disease activity. Bonferroni correction was used to adjust for multiple comparisons in four subgroups to correct the type I error. Propensity score matching was used to balance age and gender distribution among the patients in four muscle function category subgroups (in a 1:1:1:1 matching ratio), and the Friedman M test was used to compare their disease characteristics, BC, muscle function, and activity function. Chi-squared tests or Fisher’s exact tests were used for comparisons of categorical variables among groups. A polynomial contrast procedure test for tend was used in continuous variables, and the Cochran–Armitage test for trend was used in categorical data. Univariate and multivariate multinominal logistic regression analyses by calculating odds ratio (OR) and 95% confidence interval (CI) were used to identify the associations of BC, muscle function, and activity function (as independent variables) among patients with RA in subgroups according to age and disease activity (as a dependent variable). Potential confounders were adjusted including gender, smoking habits, disease duration, rheumatoid factor (RF) status, anti-cyclic citrullinated peptide antibody (ACPA) status, mTSS, comorbidities, and previous medications. All significance tests were two-tailed and were conducted at the 5% significance level.

## 3. Results

### 3.1. Demographic and Clinical Characteristics of Patients with RA

Among 1125 patients with RA recruited in the cross-sectional study, 197 were excluded for various reasons: 28 overlapped with other autoimmune diseases, 17 were accompanied with malignancy, 11 overlapped with severe liver/renal disease, 57 were without complete BC data, and 84 were without complete muscle function measurement. In total, 928 patients with RA were eligible for analysis (Figure S1), and their disease characteristics are shown in Table 1. Their mean age was 53.2 ± 12.0 years, and 771 (83.1%) were female. The median disease duration was 84 months (range 1–749 months). There were 33.3% of patients with RA in remission, 30.9% in low disease activity, 22.6% in moderate disease activity, and 13.2% in high disease activity defined by CDAI. And 22.7% of the patients were treatment naïve, defined as having no previous glucocorticoid or disease-modifying anti-rheumatic drugs (DMARDs) therapy for 6 months before enrollment.

### 3.2. Activity Function in Eight Subdimensions in Patients with RA

**Figure 1:**
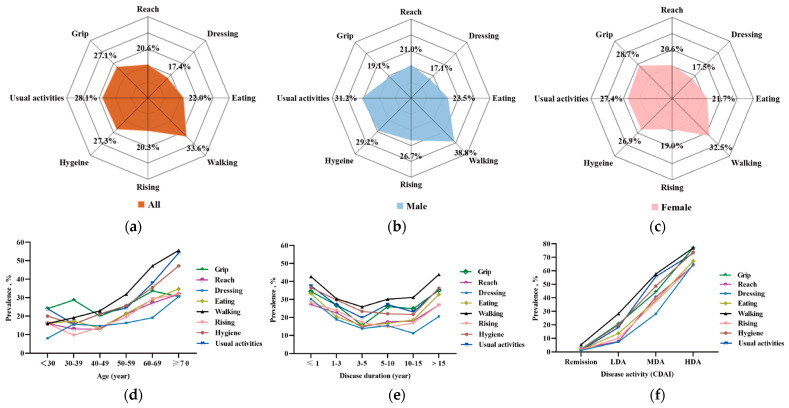
Activity function in eight subdimensions in patients with RA. (**a**) The prevalence of functional limitation in eight subdimensions among all patients; (**b**) the prevalence of functional limitation in eight subdimensions among male patients; (**c**) the prevalence of functional limitation in eight subdimensions among female patients; (**d**) the prevalence of functional limitation in eight subdimensions among all patients stratified by age; (**e**) the prevalence of functional limitation in eight subdimensions among all patients stratified by disease duration; (**f**) the prevalence of functional limitation in eight subdimensions among all patients stratified by disease activity with CDAI. CDAI, clinical disease activity index; remission, CDAI ≤ 2.8; LDA, low disease activity, 2.8 < CDAI ≤ 10.0; MDA, moderate disease activity, 10.0 < CDAI ≤ 22.0; HAD, high disease activity, CDAI > 22.0.

There were 485 (52.2%) patients with functional limitations, and the activity functions in eight subdimensions in patients with RA are shown in Figure 1. In all RA patients, the prevalence of functional limitation in upper-limb subdimensions ranged from 17.4% for dressing to 27.1% for grip. In lower-limb subdimensions, the prevalence ranged from 20.3% for rising to 33.6% for walking (Figure 1a). In male RA patients, the prevalence ranged from 17.1% for dressing to 23.5% for eating in upper-limb subdimensions, and ranged from 26.7% for rising to 38.8% for walking in lower-limb subdimensions (Figure 1b). In female RA patients, the prevalence ranged from 17.5% for dressing to 28.7% for grip in upper-limb subdimensions, and ranged from 19.0% for rising to 32.5% for walking in lower-limb subdimensions (Figure 1c).

In all RA patients, further age stratification analysis showed that the prevalence of functional limitation among eight subdimensions demonstrated different patterns (Figure 1d). In upper-limb subdimensions, the prevalence for grip fluctuated and increased, with the lowest prevalence of 20.0% at 40–49 years old and the highest prevalence of 33.6% at 60–69 years old. The prevalence for dressing initially increased from 8.0% (age < 30 years old) to a plateau between 14.7% and 16.3% (30–59 years old), and then increased to 30.6% (age ≥ 70 years old). For reach and eating, the prevalence initially decreased, reaching 12.9% for reach and 13.5% for eating at 30–49 years old, and then increased, reaching 31.9% for reach and 34.7% for eating at age ≥ 70 years old. In lower-limb subdimensions, the prevalence of functional limitation for walking increased with age from 16.0% to 55.6%. For rising, hygiene, and usual activities, the prevalence initially decreased at 30–49 years old, with the lowest prevalence ranging from 9.6% to 15.7%, and then increased, with the highest prevalence ranging from 31.9% to 54.2%. In disease duration stratification, the prevalence in these eight subdimensions showed U-shaped curves, with the bottom prevalence at 11.2–25.9% and the peak prevalence at 27.1–43.9% (Figure 1e). In disease activity stratification, the prevalence in all eight subdimensions increased sharply from 1.0–5.2% to 63.9–77.0% with disease activity (Figure 1f). In previous medication stratification, RA patients were divided into three groups: treatment naïve, glucocorticoids/csDMARDs, and bDMADRs/tsDMARDs. The prevalence in all eight subdimensions showed the lowest prevalence from 14.2% to 29.4% in the glucocorticoids/csDMARDs group (Figure S1).

### 3.3. Muscle Mass and Function in Patients with RA

**Figure 2:**
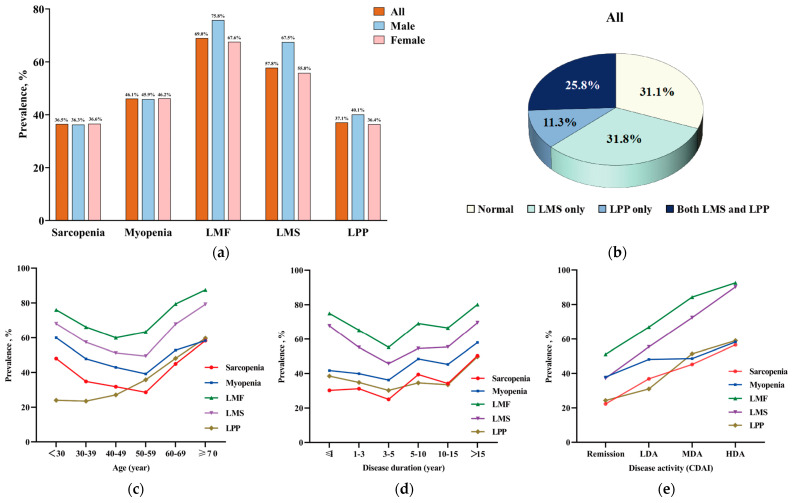
The prevalence of low muscle mass and function in patients with RA. (**a**) The prevalence of low muscle mass and function in RA patients; (**b**) the proportion of RA patients divided into four subgroups according to muscle function category; (**c**) the prevalence of low muscle mass and function in RA patients stratified by age; (**d**) the prevalence of low muscle mass and function in RA patients stratified by disease duration; (**e**) the prevalence of low muscle mass and function in RA patients stratified by disease activity with CDAI. LMF, low muscle function; LMS, low muscle strength; LPP, low physical performance; CDAI, clinical disease activity index; remission, CDAI ≤ 2.8; LDA, low disease activity, 2.8 < CDAI ≤ 10.0; MDA, moderate disease activity, 10.0 < CDAI ≤ 22.0; HAD, high disease activity, CDAI > 22.0.

The prevalence of low muscle mass and function among RA patients is shown in Figure 2. There were 339 (36.5%) RA patients with sarcopenia, 428 (46.1%) with myopenia, 640 (69.0%) with LMF, 536 (57.8%) with LMS, and 344 (37.1%) with LPP. Male and female RA patients showed a similar prevalence of low muscle mass and function compared to all RA patients. In age stratification, the prevalence of sarcopenia, myopenia, LMF, and LMS initially decreased to around 50 years old (the lowest prevalence was 28.6–63.3%), followed by an increase with advancing age (the highest prevalence was 58.3–87.5%). The prevalence of LPP increased with age from 23.5% to 59.7%, which was similar to the tendency of the prevalence of functional limitation for walking. Moreover, for muscle function assessment, in patients with RA under 50 years old, the prevalence of LMS was higher than LPP (49.4–68.0% vs. 23.5–35.8%). However, in those over 50 years old, LMS and LPP showed approximate prevalence. In disease duration stratification, the prevalence of sarcopenia, myopenia, LMF, LMS, and LPP showed W-shaped curves, with the bottom prevalence being 25.0–55.2% at 3–5 years of disease duration and the peak prevalence being 49.7–80.1% at disease duration of ≥ 15 years. In disease activity stratification, the prevalence of sarcopenia, myopenia, LMF, LMS, and LPP increased from 22.3–51.1% to 56.6–92.6% during deteriorating disease activity, which was similar to the trend in the prevalence of functional limitation. In previous medication stratification, the prevalence of sarcopenia (30.8–40.1%), myopenia (38.9–48.8%), LMF (66.4–74.7%), LMS (54.6–63.9%), and LPP (48.2–51.4%) showed no significant difference among treatment-naïve, glucocorticoids/csDMARDs, and bDMADRs/tsDMARDs groups (Figure S1).

### 3.4. Comparisons of Characteristics Among Patients with RA in Muscle Function Categorizations

According to muscle strength and physical performance, there were 289 (31.1%) patients in the normal subgroup, 295 (31.8%) patients with LMS only, 105 (11.3%) patients with LPP only, and 239 (25.8%) patients with both LMS and LPP (Figure 2b). After propensity score matching of these four subgroups by gender and age, there were 103 patients with normal muscle function, 105 patients with LMS only, 105 patients with LPP only, and 103 patients with both LMS and LPP. Comparisons of disease characteristics among RA patients in muscle function categorizations are shown in Table 2, and comparisons of body composition, muscle function, and activity function are shown in Table S1. Compared with the other three subgroups, respectively, patients with both LMS and LPP were shown to have the highest core disease activity indicators [including 28TJC, 28SJC, PtGA, PrGA, DAS28-ESR, SDAI, and CDAI (median 15 vs. 3–8)] and the worst activity function [including highest HAQ-DI, highest prevalence of functional limitation (82.5% vs. 26.2–57.1%), and for rising, eating, walking, hygiene, and usual activities] among the four subgroups. Compared with the LPP only subgroup, the LMS only subgroup had higher 28TJC, 28SJC, DAS28-CRP, SDAI, and CDAI (median 8 vs. 4), lower BMI and muscle mass indicators (including ASMI, trunk muscle, and upper-limb muscle), and higher prevalence of functional limitation in upper-limb subdimensions such as dressing, eating, and gripping (all *p* < 0.0083, Table 2 and Table S1), while there was no significant difference in the prevalence of functional limitation in lower-limb subdimensions.

### 3.5. Characteristics of Patients with RA in Subgroups According to Age and Disease Activity

**Figure 3:**
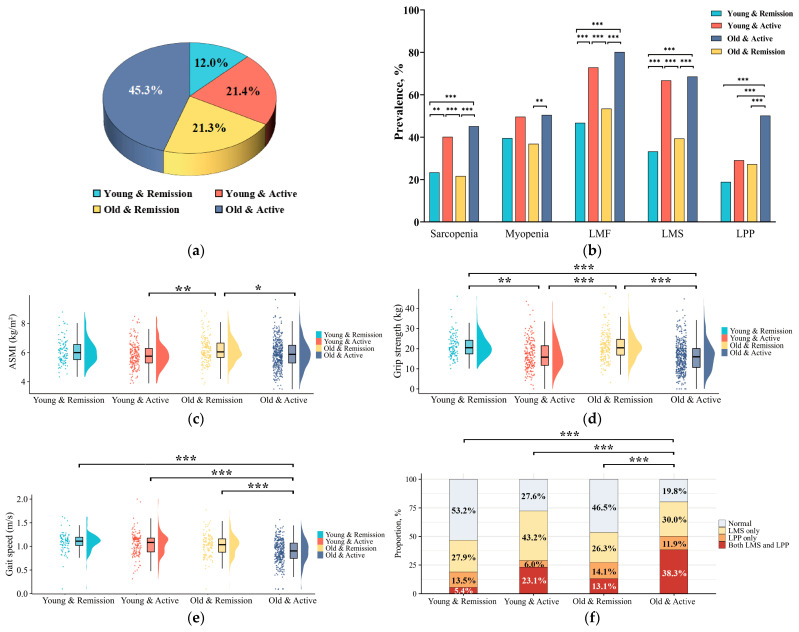
Muscle mass, muscle function, and activity function among patients with RA in subgroups according to age and disease activity. (**a**) The proportion of RA patients divided into four subgroups according to age and disease activity; (**b**) the prevalence of low muscle mass and function in these four subgroups; (**c**) comparisons of ASMI among these four subgroups; (**d**) comparisons of grip strength among these four subgroups; (**e**) comparisons of gait speed among these four subgroups; (**f**) comparisons of muscle function category among these four subgroups. Young, <50 years old; old, ≥50 years old; remission, CDAI ≤ 2.8; active, CDAI > 2.8; LMF, low muscle function; LMS, low muscle strength; LPP, low physical performance; ASMI, appendicular skeletal muscle mass index. * *p* < 0.05, ** *p* < 0.01, *** *p* < 0.001.

According to the trends of muscle mass and function in age and disease activity, respectively, patients with RA were divided into four subgroups: 111 (12.0%) patients were young (age < 50 years) and in remission (young and remission), 199 (21.4%) patients were young and had active disease (young and active), 198 (21.3%) patients were old (age ≥ 50 years) and in remission (old and remission), and 420 (45.3%) patients were old and had active disease (old and active, Figure 3a). Compared with the other three subgroups, respectively, RA patients in the old and active subgroup had the slowest gait speed, the highest prevalence of LPP (50.2% vs. 18.9–29.1%), the worst muscle function category with the highest prevalence of both LMS and LPP (38.3% vs. 5.4–23.1%), and the worst activity function [including highest HAQ-DI, highest prevalence of functional limitation (77.6% vs. 6.3–62.3%), and for rising, eating, walking, hygiene, reach, and usual activities (all *p* < 0.0083, Figure 3 and Tables S2 and S3)].

Further focusing on patients in the young and active subgroup, compared with the two remission (young and remission; old and remission) subgroups, respectively, it was surprising that they had significantly lower grip strength, higher prevalence of sarcopenia (40.2% vs. 23.4% vs. 21.7%), LMF (72.9% vs. 46.8% vs. 53.5%), and LMS (66.8% vs. 33.3% vs. 39.4%), worse muscle function category with the higher proportions of LMS only group (43.2% vs. 27.9% vs. 26.3%), as well as both the LMS and LPP groups (23.1% vs. 5.4% vs. 13.1%), and worse activity function [including higher HAQ-DI, higher prevalence of functional limitation (62.3% vs. 6.3% vs. 14.1%), and in all eight subdimensions]. They also showed significantly lower BMI and muscle mass indicators (including ASMI, trunk muscle, and upper-limb muscle) than those in the old and remission subgroup (all *p* < 0.0083, Figure 3). In addition, they showed comparable grip strength, fat and muscle mass indicators, as well as the prevalence of sarcopenia, myopenia, LMF, LMS, and functional limitations in dressing and grip in the old and active disease subgroup.

**Table 2:** Comparisons of disease characteristics among RA patients in muscle function categorizations.

Disease Characteristics	Before Propensity Score Matching					After Propensity Score Matching				
	Normal (*n* = 289)	LMS Only (*n* = 295)	LPP Only (*n* = 105)	Both LMS and LPP (*n* = 239)	*p*	Normal (*n* = 103)	LMS Only (*n* = 105)	LPP Only (*n* = 105)	Both LMS and LPP (*n* = 103)	*p*
Female, *n* (%)	251 (86.9)	239 (81.0)	92 (87.6)	189 (79.1)	0.044	91 (88.3)	95 (90.5)	92 (87.6)	91 (88.3)	0.924
Age, years, mean ± SD	51.0 ± 10.6	51.5 ± 12.9	53.8 ± 10.7	57.7 ± 12.0 ^a,b,c^	<0.001	53.2 ± 10.2	53.0 ± 12.0	53.8 ± 10.7	54.4 ± 11.6	0.703
Disease duration, month, median (IQR)	72 (35, 128)	88 (35, 141)	90 (44, 160)	96 (34, 186)	0.067	66 (30, 115)	82 (27, 131)	90 (44, 160) ^a^	95 (35, 199)	0.031
Active smoking, *n* (%)	17 (5.9)	31 (10.5)	8 (7.6)	33 (13.8) ^a^	0.017	8 (7.8)	8 (7.6)	8 (7.6)	12 (11.7)	0.669
Positive RF, *n* (%)	214 (74.0)	226 (76.6)	81 (77.1)	181 (75.7)	0.878	79 (76.7)	82 (78.1)	81 (77.1)	80 (77.7)	0.996
Positive ACPA, *n* (%)	271 (93.8)	275 (93.2)	101 (96.2)	224 (93.7)	0.751	97 (94.2)	96 (91.4)	101 (96.2)	97 (94.2)	0.545
Core disease activity indicators										
28TJC, median (IQR)	0 (0, 2)	2 (0, 6) ^a^	1 (0,3)	4 (1, 9) ^a,b,c^	<0.001	1 (0, 2)	2 (0, 6) ^a^	1 (0, 3) ^b^	4 (1, 9) ^a,b,c^	<0.001
28SJC, median (IQR)	0 (0, 1)	1 (0, 3) ^a^	0 (0, 1) ^b^	2 (0, 5) ^a,b,c^	<0.001	0 (0, 1)	1 (0, 3) ^a^	0 (0, 1) ^b^	2 (0, 7) ^a,b,c^	<0.001
PtGA, median (IQR)	1 (0, 2)	2 (1, 4) ^a^	2 (0, 4)	4 (2, 6) ^a,b,c^	<0.001	1 (0, 3)	2 (1, 3)	2 (0, 4)	5 (2, 6) ^a,b,c^	<0.001
PrGA, median (IQR)	1 (0, 2)	2 (0, 3) ^a^	1 (0, 3) ^a^	4 (2, 6) ^a,b,c^	<0.001	1 (0, 2)	2 (1, 3) ^a^	1 (0, 3)	4 (2, 5) ^a,b,c^	<0.001
PainVAS, median (IQR)	1 (0, 2)	2 (0, 3) ^a^	1 (0, 3) ^a^	3 (1, 5) ^a,b,c^	<0.001	1 (0, 2)	2 (0, 3)	1 (0, 3)	3 (1, 5) ^a,c^	<0.001
ESR, mm/h, median (IQR)	18 (10, 31)	26 (16, 47) ^a^	27 (15, 40)	35 (18, 62) ^a,b^	<0.001	23 (11, 35)	28 (18, 53)	27 (15, 40)	33 (18, 60) ^a^	0.015
CRP, mg/L, median (IQR)	3.3 (3.2, 4.2)	3.6 (3.3, 10.3) ^a^	3.6 (3.3, 6.6) ^a^	5.1 (3.4, 13.2) ^a,c^	<0.001	3.3 (3.2, 4.2)	5.0 (3.4, 19.7)	3.6 (3.3, 6.6)	4.4 (3.4, 10.6) ^a^	0.017
CDAI, median (IQR)	2 (0, 7)	8 (2, 18) ^a^	4 (0, 12)	14 (7, 25) ^a,b,c^	<0.001	3 (0, 8)	8 (2, 17) ^a^	4 (0, 12) ^b^	15 (7, 26) ^a,b,c^	<0.001
Radiographic assessments										
mTSS, median (IQR)	4 (0, 11)	10 (2, 31) ^a^	7 (2, 23)	16 (4, 56) ^a^	<0.001	3 (0, 8)	10 (2, 29) ^a^	7 (2, 23)	12 (3, 53) ^a^	0.001
JSN subscore, median (IQR)	0 (0, 4)	3 (0, 17) ^a^	1 (0, 9)	6 (1, 23) ^a^	<0.001	0 (0, 2)	3 (0, 12)	1 (0, 9)	5 (0, 32) ^a^	0.007
JE subscore, median (IQR)	2 (0, 8)	6 (1, 15) ^a^	5 (0, 16)	9 (3, 30) ^a^	<0.001	2 (0, 6)	7 (5, 16)	5 (1, 16)	9 (3, 30) ^a^	0.002
Comorbidities										
Hypertension, *n* (%)	46 (15.9)	51 (17.3)	25 (23.8)	51 (21.3)	0.190	20 (19.4)	17 (16.2)	25 (23.8)	18 (17.5)	0.522
Diabetes, *n* (%)	16 (5.5)	14 (4.7)	8 (7.6)	24 (10.0)	0.077	8 (7.8)	4 (3.8)	8 (7.6)	11 (10.7)	0.309
Cardiovascular diseases, *n* (%)	2 (0.7)	9 (3.1)	4 (3.8)	11 (4.6) ^a^	0.045	1 (1.0)	2 (1.9)	4 (3.8)	6 (5.8)	0.192
Dyslipidemia, *n* (%)	71 (24.6)	63 (21.4)	28 (26.7)	62 (25.9)	0.559	27 (26.2)	23 (21.9)	28 (26.7)	26 (25.2)	0.855
Previous medications										
Treatment naïve, *n* (%)	62 (21.5)	75 (25.4)	22 (21.0)	52 (21.8)	0.614	26 (25.2)	27 (25.7)	22 (21.0)	25 (24.3)	0.851
Glucocorticoids, *n* (%)	129 (44.6)	171 (58.0) ^a^	50 (47.6)	137 (57.3) ^a^	0.003	46 (44.7)	56 (53.3)	50 (47.6)	56 (54.4)	0.446
csDMARDs, *n* (%)	256 (88.6)	236 (80.0) ^a^	95 (90.5)	192 (80.3)	0.003	91 (88.3)	83 (79.0)	95 (90.5)	80 (77.7)	0.022
bDMADRs/tsDMARDs, *n* (%)	45 (15.6)	63 (21.4)	21 (20.0)	37 (15.5)	0.192	20 (19.4)	22 (21.0)	21 (20.0)	18 (17.5)	0.935

LMS, low muscle strength; LPP, low physical performance; RF, rheumatoid factor; ACPA, anti-cyclic citrullinated peptide antibody; 28TJC, 28-joint tender joint count; 28SJC, 28-joint swollen joint count; PtGA, patient global assessment of disease activity; PrGA, provider global assessment of disease activity; Pain VAS, pain visual analogue scale; ESR, erythrocyte sedimentation rate; CRP, C-reactive protein; CDAI, clinical disease activity index; mTSS, modified total Sharp score; JSN, joint space narrowing; JE, joint erosion; DMARDs, disease-modifying anti-rheumatic drugs; treatment naïve, having no previous glucocorticoid or DMARDs therapy for 6 months before enrollment; csDMARDs, conventional synthetic DMARDs; bDMADRs, biological DMADRs; tsDMARDs, targeted-synthetic DMADRs; IQR, interquartile range. ^a^ Compared with normal patients in Bonferroni correction, *p* < 0.0083. ^b^ Compared with LMS only patients in Bonferroni correction, *p* < 0.0083. ^c^ Compared with LPP only patients in Bonferroni correction, *p* < 0.0083.

### 3.6. Associations of Muscle Mass and Function with Patients with Young and Active Disease

**Figure 4:**
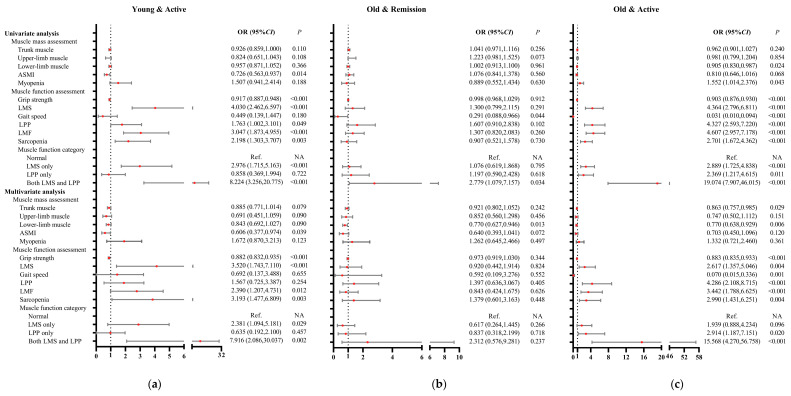
Associations of muscle mass and muscle function among patients with RA in subgroups according to age and disease activity. (**a**) Compared with the young and remission subgroup, the associations of muscle mass and muscle function in the young and active subgroup; (**b**) compared with the young and remission subgroup, the associations of muscle mass and muscle function in the old and remission subgroup; (**c**) compared with the young and remission subgroup, the associations of muscle mass and muscle function in the old and active subgroup. Multivariate analysis, adjustment for gender, smoking habits, disease duration, RF status, ACPA status, mTSS, comorbidities, and previous medications; young, <50 years old; old, ≥50 years old; remission, CDAI ≤ 2.8; active, CDAI > 2.8; ASMI, appendicular skeletal muscle mass index; LMS, low muscle strength; LPP, low physical performance; LMF, low muscle function; OR, odds ratio; CI, confidence interval; Ref, reference; NA, not applicable.

To investigate the associations of muscle mass and function among patients with RA in subgroups according to age and disease activity, univariate and multivariate multinominal logistic regression analyses were performed (Figure 4 and Table S4). Univariate multinominal logistic regression analysis showed that compared with the young and remission subgroup, the young and active subgroup was negatively associated with BMI, ASMI, and grip strength, while it was positively associated with sarcopenia, LMF, LMS, and LPP; the old and remission subgroup was negatively associated with gait speed; the old and active subgroup was negatively associated with lower-limb muscle, grip strength, and gait speed, while it was positively associated with sarcopenia, myopenia, LMF, LMS, and LPP.

After adjustment for potential confounders including gender, smoking habits, disease duration, RF status, ACPA status, mTSS, comorbidities, and previous medications, multivariate multinominal logistic regression analysis showed that compared with the young and remission subgroup, the young and active subgroup was still negatively associated with ASMI (OR = 0.606, 95%CI: 0.377–0.974) and grip strength (OR = 0.882, 95%CI: 0.832–0.935), while it was positively associated with sarcopenia (OR = 3.193, 95%CI: 1.477–6.899), LMF (OR = 2.390, 95%CI: 1.207–4.731), and LMS (OR = 3.520, 95%CI: 1.743–7.110); the old and remission subgroup was negatively associated with lower-limb muscle; the old and active subgroup was negatively associated with trunk muscle, lower-limb muscle, grip strength, and gait speed, while it was positively associated with sarcopenia (OR = 2.990, 95%CI: 1.431–6.251), LMF (OR = 3.442, 95%CI: 1.788–6.625), LMS (OR = 2.617, 95%CI: 1.357–5.046), and LPP (OR = 4.286, 95%CI: 2.108–8.715).

## 4. Discussion

This is the first study to investigate the prevalence and significance of muscle mass and function in patients with RA. A significant finding reveals that diminished muscle strength, rather than a decline in physical performance, is notably more prevalent among younger patients. Following a propensity score matching process that accounted for gender and age, the subgroup characterized by LMS only exhibited higher disease activity and reduced upper-limb muscle mass, alongside greater functional limitations (such as difficulties in dressing, eating, and gripping) compared to the subgroup with LPP only. Patients in the young and active subgroup had significantly lower grip strength, higher prevalence of sarcopenia, and worse functional limitation than the two remission (young and remission; old and remission) subgroups, respectively. They were also negatively associated with ASMI (OR of 0.606-fold) and grip strength (OR of 0.882-fold), and positively associated with sarcopenia (OR of 3.193-fold), LMF (OR of 2.390-fold), LMS (OR of 3.520-fold), and functional limitation (OR of 17.483-fold) in multivariate multinominal logistic regression analysis. This study is the first to document that individuals under the age of 50 with active RA experience compromised muscle quality and functionality, particularly within the upper extremities.

In our study, we observed the prevalence rates of sarcopenia, myopenia, LMF, LMS, and LPP among all patients with RA to be 36.5%, 46.1%, 69.0%, 57.8%, and 37.1%, respectively. Previous research has indicated that the occurrence of sarcopenia in RA patients is considerably higher compared to the general population, with prevalence estimates varying between 10.1% and 45.1% [14,15,21,22]. In other inflammatory conditions, previous studies have documented a sarcopenia prevalence of 34.3% among patients with ankylosing spondylitis [23], which is comparable to our findings, and 13.7% among those with psoriatic arthritis [24], which is lower than what we observed in our study. Moreover, a study conducted in Japan that examined 240 consecutive RA patients aged 65 years and older reported prevalence rates of 29.6% for LMF, 47.1% for LMS, and 32.9% for LPP [25], all of which were lower than those found in our study. Additionally, a recent Brazilian study involving 55 female RA patients found that 33 (60%) of the participants exhibited LMS (defined as grip strength < 16 kg) according to the EWGSOP criteria, with LPP estimates ranging from 2.4% to 40.6% [26], which revealed similar prevalence rates of LMS and LPP to our findings. Furthermore, an investigation in the UK that included 82 RA patients alongside 85 matched sedentary healthy controls demonstrated that RA patients showed significantly poorer performance in objective functional assessments, exhibiting reductions of 25.3% in handgrip strength, and 28.0% in 50-foot walking speed when compared to healthy controls [27]. The early identification of sarcopenia in RA patients is essential in mitigating adverse health outcomes. Findings from studies analyzing transcriptomic data associated with skeletal muscle outcomes in healthy older men have revealed a disconnection between muscle mass and strength [28]. Importantly, muscle strength and physical performance demonstrate a closer correlation than muscle mass with critical outcomes such as independent functioning, falls, and fractures [1].

Age serves as a significant factor influencing the probability of developing sarcopenia in otherwise healthy adults. Throughout an individual’s life, skeletal muscle strength and mass exhibit a consistent trajectory, peaking during early adulthood and subsequently experiencing a gradual decline [29]. The catabolic degradation of myofibrillar and soluble proteins via the ubiquitin–proteasome system (UPS) is considered to be the primary mechanism underlying sarcopenia [30]. Contributing factors to age-related sarcopenia include diminished protein synthesis, dysfunction of the autophagy-lysosomal system, mitochondrial impairment, and denervation of muscle fibers [31,32]. Furthermore, mitochondrial dysfunction likely plays a critical role in the onset of age-related sarcopenia, emphasizing the organelle’s essential function in the production of energy within skeletal muscle and the initiation of myocyte apoptosis [33]. Cellular senescence is another factor implicated in age-related sarcopenia. As individuals age, muscle stem cells (MuSCs) exhibit a failure to establish polarity, ultimately leading to a decrease in self-renewal capacity, enhanced differentiation, and impaired muscle regeneration [34]. However, our findings indicate that when stratifying by age, the prevalence of myopenia, LMS, LMF, and sarcopenia initially declines until approximately 50 years of age, after which it rises with advancing age. In contrast, the prevalence of LPP demonstrates a consistent increase with age. These observations suggest a distinct pattern in the changes in muscle mass and function over a lifetime between individuals with RA and healthy individuals.

Significantly, our initial findings indicate that diminished muscle strength occurs more frequently than decreased physical performance among younger individuals with RA. Notably, low grip strength serves as a significant predictor of adverse health outcomes and is commonly employed to identify sarcopenia in patients [4]. Indeed, grip strength has been found to have an inverse correlation with RA disease activity and radiographic damage in the hands and wrists, particularly with respect to joint space narrowing [35,36]. Physical performance, frequently measured by gait speed, is characterized as a comprehensive assessment encompassing cardiorespiratory fitness, neurological function, and muscle strength [4]. Nonetheless, our data reveal that, in comparison to the subgroup with LPP only, the subgroup with LMS only not only exhibited higher disease activity but also demonstrated poorer body composition. Specifically, the LMS only subgroup presented with reduced muscle mass in the trunk and upper limbs, alongside greater functional limitations in upper-limb activities, such as dressing, eating, and gripping. Interestingly, there were no notable differences in lower-limb muscle mass or functional limitations between the two subgroups. This suggests that upper-limb functionality may be more vulnerable to the combined impacts of disease activity and muscle quality than lower-limb functionality in individuals with RA.

Chronic inflammation is a defining characteristic of RA and plays a pivotal role in the progression of sarcopenia [6,37]. A meta-analysis focused on rheumatoid sarcopenia revealed that certain disease-related factors, including radiological joint damage, elevated CRP levels, and the presence of RF, significantly contribute to the risk of developing sarcopenia [15]. RA is characterized by systemic inflammation driven by various cytokines, including tumor necrosis factor (TNF)-α, interleukin (IL)-1, IL-6, and interferon-gamma (IFNγ) [3]. This heightened inflammatory state sets rheumatoid sarcopenia apart from age-related sarcopenia, a distinction that has been corroborated in both animal models of arthritis [38] and human RA patients [39]. Pro-inflammatory cytokines may not only enhance proteolysis mediated by the UPS and interfere with the self-renewal of MuSCs but could also directly impair muscle contractility by elevating the production of muscle-derived oxidants and diminishing mitochondrial content and functionality in muscle tissue [40]. Our findings corroborate these observations, indicating that as disease activity in RA patients escalates, there is a corresponding increase in the prevalence of low muscle mass and function, along with limitations in physical activity across eight distinct dimensions. This underscores the necessity for integrated management approaches that concurrently address both the disease activity of RA and the complications of sarcopenia to enhance patient outcomes.

To date, the clinical features of young and active patients with RA have garnered significant attention. These individuals have been identified as exhibiting notably poor muscle quality and functionality, particularly in the upper extremities, thus categorizing them as a high-risk population that necessitates dedicated focus. Unlike their elderly counterparts, younger RA patients bear the additional responsibilities associated with education, career obligations, familial support, and social commitments. Therefore, it is crucial to alleviate the distress experienced by young RA patients, maintain their muscle strength and functional capabilities, and support their continued engagement in learning and employment activities [41]. The factors contributing to muscle function impairment in this demographic include prolonged disease activity and the catabolic effects instigated by pro-inflammatory cytokines [6]. There remains a pressing need for further investigations to clarify the mechanisms that underlie muscle function deterioration in young and disease-active RA patients. Current management approaches for RA predominantly emphasize the control of inflammation and the prevention of joint damage, frequently neglecting the critical aspect of preserving muscle health [42]. Our findings suggest that incorporating muscle function evaluations into standard clinical protocols could facilitate the early identification of patients at high risk.

Currently, there are no drugs approved for the treatment of sarcopenia. However, resistance exercise has shown considerable evidence in enhancing strength, muscle mass, and physical function, making it a fundamental recommendation in the EWGSOP 2018 guidelines [8]. The ACR has also established guidelines advocating physical activity in individuals with RA [43]. A meta-analysis indicated that individuals suffering from joint damage, who may be incapable of performing strenuous exercises, still possess the capacity to achieve muscle hypertrophy through aerobic activities [44]. Importantly, meta-regression analysis has revealed that RA patients with a prolonged duration of the disease face greater challenges in gaining muscle compared to those diagnosed more recently [44]. Consequently, young and active RA patients may exhibit a higher likelihood of enhancing their muscle mass and functionality through aerobic exercise. Various pharmaceutical strategies aimed at addressing primary sarcopenia have been explored, including selective androgen receptor modulators [45] and the inhibition of activin receptors or myostatin through monoclonal antibodies [46]. Our previous research identified myostatin as a novel predictor of exacerbated joint deterioration in RA patients [47]. And its deficiency or antibody-mediated inhibition has been shown to mitigate inflammatory joint damage in murine models [48]. This suggests that myostatin antibodies may have promising clinical applicability in the future [49]. Additionally, in an in vitro engineered model of human skeletal muscle tissue, tofacitinib, a Janus kinase (JAK) inhibitor approved for RA treatment, effectively counteracted the adverse impacts of IFNγ on the strength, size, and architecture of skeletal muscle [50,51]. Therefore, this finding implies that there may be potential benefits for sarcopenia when tofacitinib is administered in a timely and early manner to young and active RA patients.

## 5. Limitations

Despite the merits of our investigation, which encompasses a large sample size and comprehensive data acquisition, it is imperative to recognize several limitations. Firstly, the cross-sectional nature of the study constrains our capacity to infer causal relationships between RA and the noted reductions in muscle function and mass. To substantiate these correlations, longitudinal studies are essential to elucidate the temporal progression of muscle deterioration in RA patients and to assess the potential advantages of early therapeutic interventions. Secondly, the research was exclusively conducted among Chinese patients with RA, which may restrict the applicability of the results to other demographic groups with varying genetic, environmental, and lifestyle considerations. Future studies should prioritize the inclusion of heterogeneous populations to validate and broaden our findings. Furthermore, we did not consider possible confounding variables such as nutritional status, dietary practices, physical activity levels, and socioeconomic factors, all of which could potentially impact muscle mass and function. To address these issues, future studies should include these variables, as well as larger, multi-center cohorts to enhance the robustness and applicability of the findings.

## 6. Conclusions

In conclusion, our study highlights the high prevalence of sarcopenia and reduced muscle function, particularly worse muscle strength, among RA patients, particularly in young individuals with active disease. It suggests a different trend of muscle mass and function changes throughout their lifetime between patients with RA and healthy individuals. Particularly young RA patients often serve as the backbone of their families and communities, as they carry greater responsibilities in terms of education, employment, family support, and social duties compared to older patients. These findings underscore the need for targeted clinical management strategies to address muscle issues in this population. Personalized interventions aimed at improving muscle mass and function could significantly enhance the quality of life for RA patients.

## Figures and Tables

**Table 1:** Disease characteristics of all RA patients.

Disease Characteristics	All RA (*n* = 928)
Female, *n* (%)	771 (83.1)
Age, years, mean ± SD	53.2 ± 12.0
Disease duration, month, median (IQR)	84 (36, 145)
Active smoking, *n* (%)	89 (11.2)
Positive RF, *n* (%)	702 (75.6)
Positive ACPA, *n* (%)	871 (93.9)
Core disease activity indicators	
28TJC, median (IQR)	1 (0, 5)
28SJC, median (IQR)	0 (0, 2)
PtGA, median (IQR)	2 (0, 4)
PrGA, median (IQR)	2 (0, 4)
PainVAS, median (IQR)	2 (0, 3)
ESR, mm/h, median (IQR)	25 (14, 44)
CRP, mg/L, median (IQR)	3.6 (3.3, 8.7)
CDAI, median (IQR)	7 (1, 15)
Radiographic assessments	
mTSS, median (IQR)	7 (2, 25)
JSN, median (IQR)	2 (0, 11)
JE, median (IQR)	5 (1, 14)
Comorbidities	
Hypertension, *n* (%)	173 (18.6)
Diabetes, *n* (%)	62 (6.7)
Cardiovascular diseases, *n* (%)	26 (2.8)
Dyslipidemia, *n* (%)	224 (24.1)
Previous medications	
Treatment naïve, *n* (%)	211 (22.7)
Glucocorticoids, *n* (%)	487 (52.5)
csDMARDs, *n* (%)	779 (83.9)
bDMADRs/tsDMARDs, *n* (%)	166 (17.9)

RF, rheumatoid factor; ACPA, anti-cyclic citrullinated peptide antibody; 28TJC, 28-joint tender joint count; 28SJC, 28-joint swollen joint count; PtGA, patient global assessment of disease activity; PrGA, provider global assessment of disease activity; Pain VAS, pain visual analogue scale; ESR, erythrocyte sedimentation rate; CRP, C-reactive protein; CDAI, clinical disease activity index; mTSS, modified total Sharp score; JSN, joint space narrowing; JE, joint erosion; DMARDs, disease-modifying anti-rheumatic drugs; treatment naïve, having no previous glucocorticoid or DMARDs therapy for 6 months before enrollment; csDMARDs, conventional synthetic DMARDs; bDMADRs, biological DMADRs; tsDMARDs, targeted-synthetic DMADRs; IQR, interquartile range.

## Data Availability

The original contributions presented in this study are included in the article/Supplementary Material. Further inquiries can be directed to the corresponding authors.

## Supplementary Materials

The following supporting information can be downloaded at: https://www.mdpi.com/article/10.3390/medicina61040551/s1, Figure S1: Flow chart of the study; Table S1: Comparisons of body composition, muscle function and activity function among RA patients in muscle function categorizations; Table S2: Comparisons of disease characteristics among RA patients in subgroups according to age and disease activity; Table S3: Comparisons of body composition, muscle function and activity function among RA patients in subgroups according to age and disease activity; Table S4: Associations of BMI, fat mass assessment, and activity function among patients with RA in subgroups according to age and disease activity.

## Abbreviations

The following abbreviations are used in this manuscript:

28TJC	28-joint tender joint count
28SJC	28-joint swollen joint count
ACPA	Anti-cyclic citrullinated peptide antibody
ACR	American College of Rheumatology
ASMI	Appendicular skeletal muscle mass index
AWGS	Asian Working Group for Sarcopenia
BC	Body composition
bDMADRs	Biological DMADRs
BMI	Body mass index
CDAI	Clinical disease activity index
CI	Confidence interval
CRP	C-reactive protein
csDMARDs	Conventional synthetic DMARDs
DAS28-CRP	Disease Activity Score in 28 joints including C-reactive protein
DAS28-ESR	Disease Activity Score in 28 joints including erythrocyte sedimentation rate
DMARDs	Disease-modifying anti-rheumatic drugs
ESR	Erythrocyte sedimentation rate
EULAR	European League Against Rheumatism
EWGSOP	European Working Group on Sarcopenia in Older People
HAD	High disease activity
HAQ-DI	Health assessment questionnaire disability index
IFNγ	Interferon-gamma
IL	Interleukin
IQR	Interquartile range
JAK	Janus kinase
JE	Joint erosion
JSN	Joint space narrowing
LDA	Low disease activity
LMF	Low muscle function
LMS	Low muscle strength
LPP	low physical performance
MDA	Moderate disease activity
mTSS	Modified total Sharp score
MuSCs	Muscle stem cells
NA	Not applicable
OR	Odds ratio
Pain VAS	Pain visual analogue scale
PrGA	Provider global assessment of disease activity
PtGA	Patient global assessment of disease activity
RA	Rheumatoid arthritis
Ref	Reference
RF	Rheumatoid factor
SDAI	Simplified disease activity index
TNF	Tumor necrosis factor
tsDMARDs	Targeted-synthetic DMADRs
UPS	Ubiquitin–proteasome system
