# Supplementary material for: The Characteristic of Muscle Function for Sarcopenia in Patients with Rheumatoid Arthritis: A Large-Scale Real-World Cross-Sectional Study
Source: Medicina (Kaunas). 2025 Mar 21;61(4):551. doi: 10.3390/medicina61040551 (PMC12028369; doi:10.3390/medicina61040551)
Supplement: Supplementary file 1 [file medicina-61-00551-s001.zip › Supplemental file-Figure S1-revised.pdf]

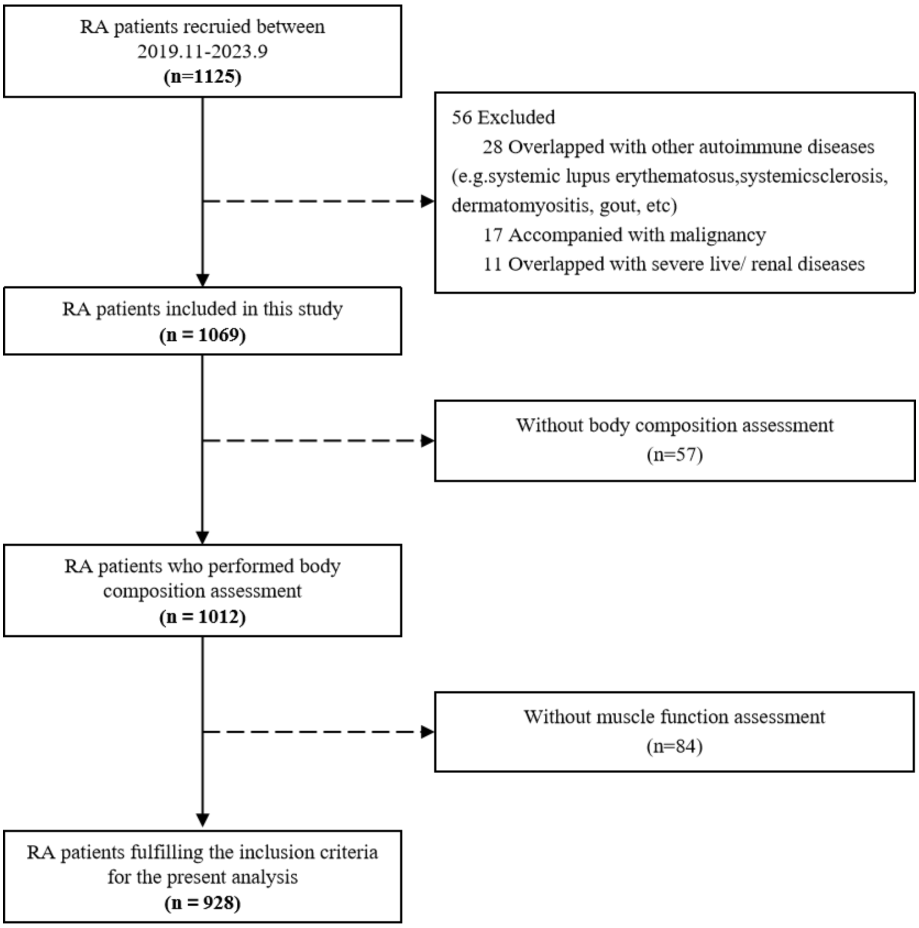

(**a**)

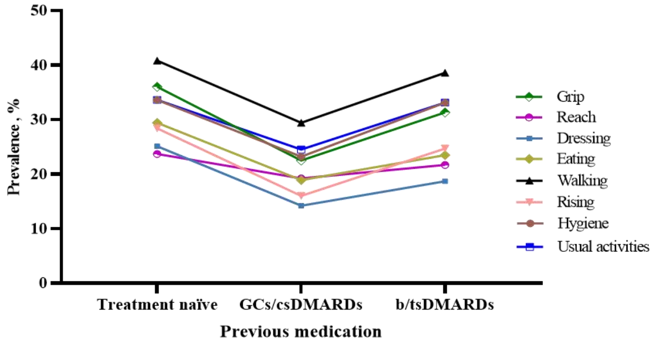

(**b**)

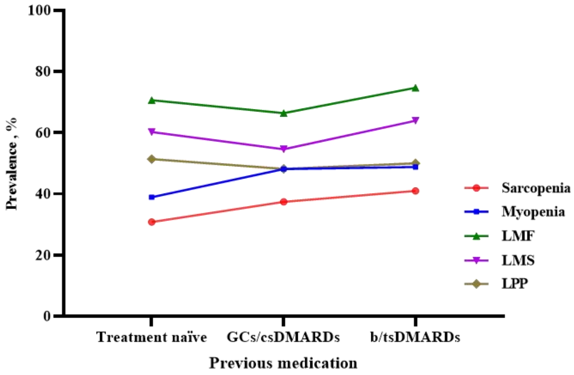

(**c**)

Figure S1. Supplementary figures of the study. (**a**) Flow chart of the study; (**b**) Activity function in eight subdimensions in RA patients stratified by previous medications; (**c**) The prevalence of low muscle mass and function in RA patients stratified by previous medications. LMF, low muscle function; LMS, low muscle strength; LPP, low physical performance.
