# Supplementary material for: The Characteristic of Muscle Function for Sarcopenia in Patients with Rheumatoid Arthritis: A Large-Scale Real-World Cross-Sectional Study
Source: Medicina (Kaunas). 2025 Mar 21;61(4):551. doi: 10.3390/medicina61040551 (PMC12028369; doi:10.3390/medicina61040551)
Supplement: Supplementary file 1 [file medicina-61-00551-s001.zip › Supplemental file-Tables.pdf]

Table S1. Comparisons of body composition, muscle function and activity function among RA patients in muscle function categorizations.

Characteristics	Before propensity score matching					After propensity score matching				
	**Normal (n=289)**	**LMS only (n=295)**	**LPP only (n=105)**	**Both LMS and LPP (n=239)**	***P***	**Normal (n=103)**	**LMS only (n=105)**	**LPP only (n=105)**	**Both LMS and LPP (n=103)**	***P***
Body composition										
BMI, kg/m^2, mean±SD	22.4±3.0	21.3±3.2[a]	22.6±3.8[b]	21.6±3.2[a,c]	**<0.001**	22.8±3.0	21.5±3.2[a]	22.6±3.8[b]	22.0±3.3	**0.009**
Fat mass assessment										
Fat mass, kg, mean±SD	16.8±5.6	15.7±5.8	17.2±7.0	16.1±6.5	0.061	17.4±5.9	16.4±6.4	17.2±7.0	17.0±6.4	0.545
Trunk fat, kg, mean±SD	8.2±3.0	7.6±3.2	8.4±3.8	7.7±3.5	**0.036**	8.6±3.1	7.9±3.5	8.4±3.8	8.2±3.5	0.402
Upper-limb fat, kg, mean±SD	2.3±1.0	2.2±1.0	2.4±1.2	2.3±1.1	0.312	2.4±1.0	2.4±1.2	2.4±1.2	2.4±1.1	0.615
Lower-limb fat, kg, mean±SD	5.3±1.6	5.0±1.6	5.4±2.0	5.1±1.9	0.077	5.4±1.7	5.2±1.8	5.4±2.0	5.3±1.8	0.772
BF%, %, mean±SD	29.3±7.0	28.8±8.0	29..7±8.3	29.7±9.1	0.599	30.1±7.2	30.2±9.0	29.7±8.3	30.8±8.7	0.412
Muscle mass assessment										
Trunk muscle, kg, mean±SD	17.5±3.1	16.4±3.2[a]	17.2±2.8	15.9±3.1[a,c]	**<0.001**	17.7±3.3	15.9±2.5[a]	17.2±2.8[b]	16.0±2.8[a,c]	**<0.001**
Upper-limb muscle, kg, mean±SD	3.8±1.0	3.5±1.0[a]	3.7±0.9	3.3±1.0[a,c]	**<0.001**	3.9±1.0	3.3±0.8[a]	3.7±0.9[b]	3.3±0.9[a,c]	**<0.001**
Lower-limb muscle, kg, mean±SD	12.0±2.2	11.4±2.5[a]	11.8±2.2	10.9±2.5[a,c]	**<0.001**	11.8±1.9	10.9±2.0	11.8±2.2	10.9±2.1[a,c]	**<0.001**
ASMI, kg/m^2, mean±SD	6.2±0.8	5.9±0.9[a]	6.2±0.8[b]	5.7±1.0[a,c]	**<0.001**	6.3±0.8	5.7±0.8[a]	6.2±0.8[b]	5.8±0.9[a,c]	**<0.001**
Myopenia, n (%)	89 (30.8)	151 (51.2)[a]	36 (34.3)[b]	152 (63.6)[a,b,c]	**<0.001**	29 (28.2)	51 (48.6)	36 (34.3)	57 (55.3)[a]	**<0.001**
Muscle function assessment										
Grip strength, kg, mean±SD	24.4±6.5	14.2±5.3[a]	24.3±8.1[b]	12.9±5.1[a,c]	**<0.001**	24.5±6.0	13.1±4.8[a]	24.3±8.1[b]	12.3±4.7[a,c]	**<0.001**
LMS, n (%)	0 (0)	295 (100)[a]	0 (0)[b]	239 (100)[a,c]	**<0.001**	0 (0)	105 (100)[a]	0 (0)[b]	103 (100)[a,c]	**<0.001**
Gait speed, m/s, mean±SD	1.2±0.1	1.2±0.1	0.8±0.2[a,b]	0.8±0.2[a,b]	**<0.001**	1.2±0.1	1.2±0.2	0.8±0.2[a,b]	0.8±0.2[a,b]	**<0.001**
LPP, n (%)	0 (0)	0 (0)	105 (100)[a,b]	239 (100)[a,b]	**<0.001**	0 (0)	0 (0)	105 (100)[a,b]	103 (100)[a,b]	**<0.001**
LMF, n (%)	0 (0)	295 (100)[a]	105 (100)[a]	239 (100)[a]	**<0.001**	0 (0)	105 (100)[a]	105 (100)[a]	103 (100)[a]	**<0.001**
Sarcopenia, n (%)	0 (0)	151 (51.2)[a]	36 (34.3)[a,b]	152 (63.6)[a,b,c]	0.599	0 (0)	51 (48.6)[a]	36 (34.3)[a]	57 (55.3)[a]	**<0.001**
Activity function										
HAQ-DI, median (IQR)	0 (0,0)	0.1 (0,0.5)[a]	0 (0,0.4)[a]	0.6 (0.1,1.1)[a,b,c]	**<0.001**	0 (0,0.1)	0.1 (0,0.4)[a]	0 (0,0.4)[a]	0.6 (0.1,1.1)[a,b,c]	**<0.001**

Functional limitation, n (%)	68 (23.5)	170 (57.6)[a]	50 (47.6)[a]	197 (82.4)[a,b,c]	**<0.001**	27 (26.2)	60 (57.1)[a]	50 (47.6)	85 (82.5)[a,b,c]	**<0.001**
Grip, (%)	22 (7.6)	96 (32.5)[a]	17 (16.2)[b]	117 (49.0)[a,b,c]	**<0.001**	9 (8.7)	31 (29.5)	17 (16.2)[b]	54 (52.4)[a,c]	**<0.001**
Reach, (%)	19 (6.6)	59 (20.0)[a]	14 (13.3)	100 (41.8)[a,b,c]	**<0.001**	6 (5.8)	21 (20.0)	14 (13.3)	42 (40.8)[a,c]	**<0.001**
Dressing, (%)	13 (4.5)	63 (21.4)[a]	5 (4.8)[b]	81 (33.9)[a,b,c]	**<0.001**	7 (6.8)	23 (21.9)	5 (4.8)[b]	34 (33.0)[a,c]	**<0.001**
Eating, (%)	18 (6.2)	75 (25.4)[a]	9 (8.6)[b]	103 (43.1)[a,b,c]	**<0.001**	11 (10.7)	26 (24.8)	9 (8.6)[b]	51 (49.5)[a,b,c]	**<0.001**
Walking, (%)	34 (11.8)	91 (30.8)[a]	40 (38.1)[a]	147 (61.5)[a,b,c]	**<0.001**	15 (14.6)	32 (30.5)	40 (38.1)	64 (62.1)[a,b,c]	**<0.001**
Rising, (%)	16 (5.5)	52 (17.6)[a]	16 (15.2)[a]	105 (43.9)[a,b,c]	**<0.001**	9 (8.7)	16 (15.2)	16 (15.2)	47 (45.6)[a,b,c]	**<0.001**
Hygiene, (%)	23 (8.0)	76 (25.8)[a]	24 (22.9)[a]	131 (54.8)[a,b,c]	**<0.001**	10 (9.7)	26 (24.8)	24 (22.9)	55 (53.4)[a,b,c]	**<0.001**
Usual activities, (%)	16 (5.5)	71 (24.1)[a]	31 (29.5)[a]	143 (59.8)[a,b,c]	**<0.001**	6 (5.8)	24 (22.9)	31 (29.5)	63 (61.2)[a,b,c]	**<0.001**

BMI, body mass index; BF%, body fat percentage; ASMI, appendicular skeletal muscle mass index; LMS, low muscle strength; LPP, low physical performance; LMF, low muscle function; functional limitation, HAQ-DI>0; IQR, interquartile range.

[a]Compared with normal patients in Bonferroni correction, P <0.0083.

[b]Compared with LMS only patients in Bonferroni correction, P <0.0083.

[c]Compared with LPP only patients in Bonferroni correction, P <0.0083.

Table S2. Comparisons of disease characteristics among RA patients in subgroups according to age and disease activity.

Disease characteristics	Young (<50 years old)		Old (≥50 years old)		*P*
	Remission (n=111)	Active (n=199)	Remission (n=198)	Active (n=420)	
Female, n (%)	102 (91.9)	177 (88.9)	153 (77.3)[a,b]	339 (80.7)[a]	**0.001**
Age, years, mean±SD	39.3±7.1	39.7±7.3	59.5±6.8[a,b]	60.3±7.3[a,b]	**<0.001**
Disease duration, month, median (IQR)	75 (35,122)	75 (26,124)	83 (44,140)	97 (36,175)[b]	**0.008**
Active smoking, n (%)	3 (2.7)	12 (6.0)	28 (14.1)[a,b]	46 (11.0)[a]	**0.002**
Positive RF, n (%)	80 (72.1)	146 (73.4)	158 (79.8)	318 (75.7)	0.364
Positive ACPA, n (%)	107 (96.4)	186 (93.5)	184 (92.9)	394 (93.8)	0.661
Core disease activity indicators					
28TJC , median (IQR)	0 (0,0)	3 (1,6)[a]	0 (0,0)[b]	3 (1,8)[a,c]	**<0.001**
28SJC, median (IQR)	0 (0,0)	1 (0,4)[a]	0 (0,0)[b]	1 (0,4)[a,c]	**<0.001**
PtGA, median (IQR)	0 (0,0)	3 (1,5)[a]	0 (0,0)[b]	4 (2,5)[a,c]	**<0.001**
PrGA, median (IQR)	0 (0,0)	2 (1,4)[a]	0 (0,0)[b]	3 (2,5)[a,b,c]	**<0.001**
PainVAS, median (IQR)	0 (0,0)	2 (2,4)[a]	0 (0,0)[b]	3 (2,5)[a,c]	**<0.001**
ESR, mm/h, median (IQR)	15 (10,24)	24 (13,38)[a]	22 (12,36)[a]	33 (18,55)[a,b,c]	**<0.001**
CRP, mg/L, median (IQR)	3.3 (3.2,3.6)	3.7 (3.3,12.4)[a]	3.6 (3.3,4.3)[b]	4.3 (3.3,11.7)[a,c]	**<0.001**
CDAI, median (IQR)	0 (0,2)	10 (6,19)[a]	0 (0,1)[b]	12 (7,21)[a,c]	**<0.001**
Radiographic assessments					
mTSS, median (IQR)	4 (0,12)	8 (1,31)[a]	5 (0,12)	11 (4,48)[a,c]	**<0.001**
JSN, median (IQR)	1 (0,6)	3 (0,17)	0 (0,4)[b]	4 (0,20)[a,c]	**<0.001**
JE, median (IQR)	2 (0,6)	4 (1,14)	4 (1,9)	7 (2,25)[a,c]	**<0.001**
Comorbidities					
Hypertension, n (%)	4 (3.6)	10 (5.0)	59 (29.8)[a,b]	100 (23.8)[a,b]	**<0.001**
Diabetes, n (%)	1 (0.9)	4 (2.0)	23 (11.6)[a,b]	34 (8.1)[a,b]	**<0.001**
Cardiovascular diseases, n (%)	0 (0)	1 (0.5)	6 (3.0)	19 (4.5)	**0.009**
Dyslipidemia, n (%)	14 (12.6)	38 (19.1)	56 (28.3)[a]	116 (27.6)[a]	**0.001**
Previous medications					
Treatment naïve, n (%)	19 (17.1)	46 (23.1)	40 (20.2)	106 (25.2)	0.238
Glucocorticoids, n (%)	59 (53.2)	109 (54.8)	85 (42.9)	234 (55.7)[c]	**0.024**
csDMARDs, n (%)	104 (93.7)	157 (78.9)[a]	178 (89.9)	340 (81.0)[a,c]	**<0.001**
bDMADRs/tsDMARDs, n (%)	21 (18.9)	48 (26.1)	28 (15.9)[b]	58 (14.5)[b]	**0.007**

Remission, CDAI≤2.8; active, CDAI>2.8; IQR, interquartile range.

[a]Compared with young & remission subgroup in Bonferroni correction, *P* <0.0083.

[b]Compared with young & active subgroup in Bonferroni correction, *P* <0.0083.

[c]Compared with old & remission subgroup in Bonferroni correction, *P* <0.0083.

Table S3. Comparisons of body composition, muscle function and activity function among RA patients in subgroups according to age and disease activity.

Characteristics	Young (<50 years old)		Old (≥50 years old)		*P*
	Remission (n=111)	Active (n=199)	Remission (n=198)	Active (n=420)	
Body composition					
BMI, kg/m^2, mean±SD	21.8±3.3	21.0±3.3	22.2±2.9[b]	22.1±3.3[b]	**<0.001**
Fat mass assessment					
Fat mass, kg, mean±SD	16.3±5.9	15.8±5.8	16.3±5.7	16.6±6.4	0.519
Trunk fat, kg, mean±SD	7.8±3.2	7.6±3.2	8.0±3.1	8.1±3.5	0.348
Upper-limb fat, kg, mean±SD	2.2±1.0	2.2±0.9	2.2±1.0	2.3±1.1	0.241
Lower-limb fat, kg, mean±SD	5.3±1.7	5.1±1.6	5.1±1.6	5.2±1.8	0.728
BF%, %, mean±SD	29.1±6.8	29.1±7.7	28.8±7.9	29.7±8.5	0.498
Muscle mass assessment					
Trunk muscle, kg, mean±SD	16.9±3.5	16.3±3.1	17.4±3.0[b]	16.5±3.2[c]	**0.004**
Upper-limb muscle, kg, mean±SD	3.6±1.1	3.4±1.0	3.8±1.0[b]	3.6±1.0[c]	**0.001**
Lower-limb muscle, kg, mean±SD	11.8±1.9	11.5±2.4	11.8±2.3	11.2±2.5[c]	**0.015**
ASMI, kg/m^2, mean±SD	6.1±0.8	5.8±0.9	6.2±0.9[b]	5.9±1.0[c]	**0.001**
Myopenia, n (%)	44 (39.6)	99 (49.7)	73 (36.9)	212 (50.5)[c]	**0.005**
Muscle function assessment					
Grip strength, kg, mean±SD	21.3±5.7	16.8±7.4[a]	21.2±6.8[b]	16.1±7.3[a,c]	**<0.001**
LMS, n (%)	37 (33.3)	133 (66.8)[a]	78 (39.4)[b]	288 (68.6)[a,c]	**<0.001**
Gait speed, m/s, mean±SD	1.1±0.2	1.0±0.2	1.0±0.2	0.9±0.3[a,b,c]	**<0.001**
LPP, n (%)	21 (18.9)	58 (29.1)	54 (27.3)	211 (50.2)[a,b,c]	**<0.001**
LMF, n (%)	52 (46.8)	145 (72.9)[a]	106 (53.5)[b]	337 (80.2)[a,c]	**<0.001**
Sarcopenia, n (%)	26 (23.4)	80 (40.2)[a]	43 (21.7)[b]	190 (45.2)[a,c]	**<0.001**
Muscle function category		[a]	[b]	[a,b,c]	**<0.001**
Normal, n (%)	59 (53.2)	55 (27.6)	22 (46.5)	83 (19.8)	
LMS only, n (%)	31 (27.9)	86 (43.2)	52 (26.3)	126 (30.0)	
LPP only, n (%)	15 (13.5)	12 (6.0)	28 (14.1)	50 (11.9)	
Both LMS and LPP, n (%)	6 (5.4)	46 (23.1)	26 (13.1)	161 (38.3)	
Activity function					
HAQ-DI, median (IQR)	0 (0,0)	0.1 (0,0.5)[a]	0 (0,0)[b]	0.4 (0.1,0.9)[a,b,c]	**<0.001**
Functional limitation, n (%)	7 (6.3)	124 (62.3)[a]	28 (14.1)[b]	326 (77.6)[a,b,c]	**<0.001**
Grip, (%)	2 (1.8)	71 (35.7)[a]	4 (2.0)[b]	175 (41.7)[a,c]	**<0.001**
Reach, (%)	1 (0.9)	40 (20.1)[a]	6 (3.0)[b]	145 (34.5)[a,b,c]	**<0.001**
Dressing, (%)	0 (0)	45 (22.6)[a]	3 (1.5)[b]	114 (27.1)[a,c]	**<0.001**
Eating, (%)	0 (0)	47 (23.6)[a]	4 (2.0)[b]	154 (36.7)[a,b,c]	**<0.001**
Walking, (%)	3 (2.7)	62 (31.2)[a]	13 (6.6)[b]	234 (55.7)[a,b,c]	**<0.001**
Rising, (%)	0 (0)	39 (19.6)[a]	4 (2.0)[b]	146 (34.8)[a,b,c]	**<0.001**
Hygiene, (%)	1 (0.9)	58 (29.1)[a]	4 (2.0)[b]	191 (45.5)[a,b,c]	**<0.001**
Usual activities, (%)	0 (0)	60 (30.2)[a]	3 (1.5)[b]	198 (47.1)[a,b,c]	**<0.001**

Remission, CDAI≤2.8; active, CDAI>2.8; BMI, body mass index; BF%, body fat percentage; ASMI, appendicular

skeletal muscle mass index; LMS, low muscle strength; LPP, low physical performance; LMF, low muscle function; functional limitation, HAQ-DI>0; IQR, interquartile range.

[a]Compared with young & remission subgroup in Bonferroni correction, P <0.0083.

[b]Compared with young & active subgroup in Bonferroni correction, P <0.0083.

[c]Compared with old & remission subgroup in Bonferroni correction, P <0.0083.

Table S4. Associations of BMI, fat mass assessment, and activity function among patients with RA in subgroups according to age and disease activity.

**Characteristics**	**Young (<50 years old)**				**Old (≥50 years old)**			
	**Remission (n=111)**		**Active (n=199)**		**Remission (n=198)**		**Active (n=420)**	
	***OR* (*95%CI*)**	***P***	***OR* (*95%CI*)**	***P***	***OR* (*95%CI*)**	***P***	***OR* (*95%CI*)**	***P***
**Univariate analysis**								
Body composition								
BMI, kg/m²	Ref.	NA	0.928 (0.861,0.999)	**0.008**	1.043 (0.970,1.122)	0.256	1.038 (0.972,1.109)	0.265
Fat mass assessment								
Fat mass, kg	Ref.	NA	0.989 (0.951,1.028)	0.560	1.001 (0.963,1.040)	0.953	1.010 (0.976,1.046)	0.575
Trunk fat, kg	Ref.	NA	0.981 (0.914,1.053)	0.595	1.017 (0.948,1.092)	0.639	1.027 (0.964,1.095)	0.407
Upper-limb fat, kg	Ref.	NA	0.983 (0.780,1.238)	0.883	0.990 (0.785,1.247)	0.930	1.127 (0.918,1.384)	0.254
Lower-limb fat, kg	Ref.	NA	0.943 (0.825,1.078)	0.387	0.947 (0.828,1.082)	0.421	0.982 (0.871,1.106)	0.763
BF%, %	Ref.	NA	1.000 (0.971,1.029)	0.981	0.994 (0.966,1.023)	0.697	1.010 (0.984,1.037)	0.467
Activity function								
HAQ-DI	Ref.	NA	1.044×10^{7} (3.517×10^{4},3.098×10^{9})	**<0.001**	9.495×10^{3} (28.508,3.163×10^{6})	**<0.001**	2.161×10^{7} (7.292×10^{4},6.404×10^{9})	
Functional limitation	Ref.	NA	24.564 (1.0848,55.620)	**<0.001**	2.447 (1.032,5.803)	**0.042**	51.526 (23.176,114.556)	**<0.001**
Grip	Ref.	NA	30.230 (7.247,126.112)	**<0.001**	1.124 (0.203,6.235)	0.894	38.929 (9.486,159.761)	**<0.001**
Reach	Ref.	NA	27.673 (3.748,204.302)	**0.001**	3.437 (0.409,28.924)	0.256	58.000 (8.015,419.702)	**<0.001**
Dressing	Ref.	NA	1.386×10^{8} (9.328×10^{7},2.058×10^{8})	**<0.001**	7.295×10^{6} (2.286×10^{6},2.328×10^{7})	**<0.001**	1.766×10^{8} (1.766×10^{8},1.766×10^{8})	**<0.001**
Eating	Ref.	NA	1.186×10^{8} (8.091×10^{7},1.739×10^{8})	**<0.001**	7.910×10^{6} (2.882×10^{6},2.171×10^{7})	**<0.001**	2.221×10^{8} (2.221×10^{8},2.221×10^{8})	**<0.001**
Walking	Ref.	NA	16.292 (4.977,53.327)	**<0.001**	2.530 (0.705,9.077)	0.155	45.290 (14.152,144.942)	**<0.001**
Rising	Ref.	NA	1.029×10^{8} (6.872×10^{7},1.540×10^{8})	**<0.001**	8.702×10^{6} (3.169×10^{6},2.390×10^{7})	**<0.001**	2.249×10^{8} (2.249×10^{8},2.249×10^{8})	**<0.001**
Hygiene	Ref.	NA	45.248 (6.170,331.827)	**<0.001**	2.268 (0.250,20.546)	0.466	91.747 (12.690,663.302)	**<0.001**

Usual activities	Ref.	NA	1.321×10^{8} (9.230×10^{7},1.890×10^{8})	**<0.001**	4.707×10^{6} (1.481×10^{6},1.496×10^{7})	**<0.001**	2.729×10^{8} (2.221×10^{8},2.221×10^{8})	**<0.001**
**Multivariate analysis**								
Body composition								
BMI, kg/m^2	Ref.	NA	0.930 (0.827,1.045)	0.221	0.946 (0.840,1.066)	0.364	1.001 (0.899,1.115)	0.985
Activity function								
HAQ-DI	Ref.	NA	7.654×10^{6} (3.385×10^{3},1.730×10^{10})	**<0.001**	845.245 (0.242,2.952×10^{6})	0.105	1.436×10^{7} (6.354×10^{3},3.245×10^{10})	**<0.001**
Functional limitation	Ref.	NA	17.483 (5.781,52.866)	**<0.001**	1.552 (0.445,5.405)	0.490	34.185 (11.442,102.136)	**<0.001**
Grip	Ref.	NA	3.665×10^{8} (2.066×10^{8},6.501×10^{8})	**<0.001**	8.763×10^{6} (1.172×10^{6},6.554×10^{7})	**<0.001**	5.866×10^{8} (5.866×10^{8},5.866×10^{8})	**<0.001**
Reach	Ref.	NA	13.713 (1.762,106.749)	**0.012**	1.698 (0.166,17.351)	0.655	26.638 (3.518,201.698)	**0.001**
Dressing	Ref.	NA	7.816×10^{7} (0,+∞)	0.995	3.843×10^{6} (0,+∞)	0.996	1.146×10^{8} (0,+∞)	0.995
Eating	Ref.	NA	1.692×10^{8} (8.565×10^{7},3.345×10^{8})	**<0.001**	1.846×10^{7} (4.196×10^{6},8.118×10^{7})	**<0.001**	4.241×10^{8} (4.241×10^{8},4.241×10^{8})	**<0.001**
Walking	Ref.	NA	10.863 (2.441,48.346)	**0.002**	1.228 (0.225,6.689)	0.812	21.003 (4.847,91.011)	**<0.001**
Rising	Ref.	NA	1.787×10^{8} (8.696×10^{7},3.672×10^{8})	**<0.001**	1.090×10^{7} (2.373×10^{6},5.006×10^{7})	**<0.001**	3.208×10^{8} (3.208×10^{8},3.208×10^{8})	**<0.001**
Hygiene	Ref.	NA	13.786 (1.779,106.827)	**0.012**	0.315 (0.018,5.482)	0.428	27.665 (3.665,208.855)	**0.001**
Usual activities	Ref.	NA	2.481×10^{8} (1.371×10^{8},4.490×10^{8})	**<0.001**	1.113×10^{7} (2.527×10^{6},4.900×10^{7})	**<0.001**	4.684×10^{8} (4.684×10^{8},4.684×10^{8})	**<0.001**

Remission, CDAI≤2.8; active disease, CDAI>2.8; BMI, body mass index; BF%, body fat percentage; ASMI, appendicular skeletal muscle mass index; LMS, low muscle strength; LPP, low physical performance; LMF, low muscle function; functional limitation, HAQ-DI>0; IQR, interquartile range; *OR*, odds ratio; 95%*CI*, 95% confidence interval; Ref, reference; NA, not applicable.

Multivariate analysis, adjustment for gender, smoking habits, disease duration, RF status, ACPA status, mTSS, comorbidities, and previous medications.
